# Clinical features and pathogenesis of Sjogrens disease related peripheral neuropathy and their relevance to clinical trials

**DOI:** 10.1038/s43856-026-01688-2

**Published:** 2026-06-19

**Authors:** Valentina Pucino, Saba Nayar, Janev Fehmi, Chiara Baldini, Simon J. Bowman, Benjamin A. Fisher

**Affiliations:** 1 Department of Clinical and Experimental Medicine, Santa Chiara Hospital, University of Pisa, Pisa, Italy; 2 Rheumatology Research Group, School of Infection, Inflammation and Immunology, University of Birmingham, Birmingham, UK; 3 NIHR Birmingham Biomedical Research Centre, University Hospitals Birmingham NHS Foundation Trust and University of Birmingham, Birmingham, UK; 4 Department of Neurology, North Bristol NHS Trust, Bristol, UK; 5 Milton Keynes University Hospital, Milton Keynes, UK

**Keywords:** SjÃ¶gren's disease, Neurological manifestations

## Abstract

Sjögren disease (SjD) is a chronic systemic autoimmune disorder characterized by immune-mediated destruction of moisture-producing glands (e.g. tears/saliva), leading to dryness. It also affects organs outside the glands, reflecting its systemic nature. Neurologic involvement is a common complication of SjD that can occur in up to 20% of SjD patients. Neurological manifestations in SjD span the central, peripheral, and autonomic systems, with the highest incidence of involvement occurring within the peripheral nervous system (PNS). The heterogeneity of neurologic manifestations in SjD complicates the diagnosis and treatment, which should be directed toward the underlying neuropathologic mechanism which is often unclear. Optimizing the diagnosis, evaluation, and management of these manifestations is essential to prevent severe disability and to design more effective clinical trials. In this review, we summarize the current understanding of SjD-related peripheral neuropathies. By detailing their specific pathogenetic mechanisms, we advocate for a targeted diagnostic and therapeutic framework designed to improve long-term patient outcomes.

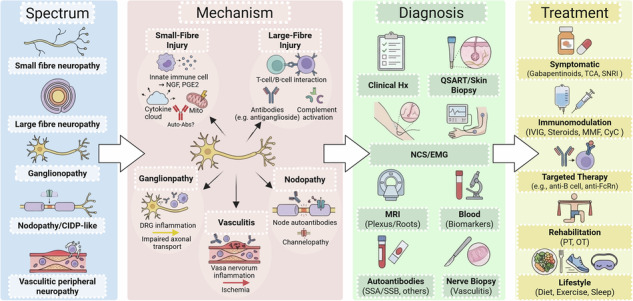

## Abstract (web summary)

Pucino et al. examine the clinical challenges of Sjögren’s disease, a systemic autoimmune disorder frequently associated with peripheral neurological manifestations. This review explores shared and distinct pathogenic mechanisms and discusses strategies to improve diagnosis, management, and future therapeutic development.

## Introduction

SjD manifests as a systemic autoimmune disease, where chronic inflammation typically targets and disrupts the function of the mosture producing (exocrine) glands^1^. The clinical picture is mainly dominated by mucosal dryness; however, a consistent proportion of patients with SjD can also experience extra-glandular manifestations including the lungs, the kidney, the joints, the nervous system as well as haematological complications such as lymphoma^1^. The risk of B-cell lymphoma is 15 to 20 times higher among patients with SjD than in the general population (lifetime risk, 5–10%)^1,2^.

SjD can occur alone (primary SjD) or in conjunction with other systemic connective tissue diseases (associated’ SjD) such as Rheumatoid Arthritis (RA), Systemic Lupus Erythematous (SLE) and Systemic Sclerosis (SSc)^3–5^. Pathogenetically, the disease is characterized by innate and adaptive immune dysregulation, which triggers B cell activation and the subsequent production of autoantibodies^3^. The classification of SjD is currently based on the 2016 ACR/EULAR criteria (Table 1)^6^. Anti Ro60 antibodies seem more specifically associated with SjD in comparison with isolated anti-Ro52 antibodies which are observed in a broad range of connective tissue diseases^7^. However, the presence of both Ro60/52 together may associated with more severe disease^8^. Labial salivary gland (LSG) biopsy is used in the diagnosis and classification of SjD and may also function as a biomarker of future lymphoma risk. However, it is important to be aware that the acquisition of tissue and histological interpretation may be variable between centres and observers, and this may confound accurate diagnosis. Consensus recommendations for standardisation have been published^9^ and more recently we have published data regarding the minimum amount of salivary gland tissue required for measurement accuracy^10^. Although not included in the ACR/EULAR 2016 classification criteria themselves, extra-glandular systemic manifestations such as neurological involvement may serve as entry criteria for their application, supporting the clinical suspicion of SjD^10^.

**Table 1:** ACR-EULAR Classification Criteria for primary SjD

Parameter	Weight/Score
Labial salivary gland with focal lymphocytic sialadenitis and focus score ≥1.3	3
Anti-SSA (Ro)+	3
Ocular staining score ≥5 (or van Bijsterfeld score ≥4) on at least one eye	1
Schirmer ≤5 mm/5 min on at least one eye	1
Unstimulated whole saliva flow rate ≤0.1 ml/min5	1

The classification of SjD applies to any individual who meets the inclusion criteria (inclusion criteria: these criteria are applicable to any patient with at least one symptom of ocular or oral dryness or suspicion of SjD from ESSDAI questionnaire (at least one domain with positive item)), does not have any condition listed as exclusion criteria (exclusion criteria: 1) History of head and neck radiation treatment; 2) Active Hepatitis C infection (with positive PCR); 3) acquired immunodeficiency syndrome; 4) sarcoidosis; 5) amyloidosis; 6) graft versus host disease; 7) IgG4-related disease), and who has a score ≥ 4 when summing the weights from the above items. Referenced in ref. ^6^.

This review highlights the major peripheral neuropathy phenotypes in SjD, outlines shared and distinct pathogenetic mechanisms and emphasizes a mechanism-based diagnostic and therapeutic approach to improve patient outcomes and guide future clinical trials.

## Spectrum of neurological manifestations pathology in SjD

Neurological manifestations can occur in 18–45% of SjD patients^11^. The prevalence of neurological involvement reported will be influenced by the nature of the cohort studied and its referral pathways, criteria for diagnosing neurological involvement, and whether past damage from neurological involvement as well current activity is being considered. Notably, in recent larger clinical trials in systemic SjD where the frequency of peripheral nervous system (PNS) involvement is reported, the baseline prevalence of active disease in that domain varied from ~7 to 16%. Involvement of the PNS is more common than that of the central nervous system (CNS) and autonomic system^11,12^. Neurological manifestations are more likely to occur in male patients, and in association with other extra-glandular manifestations such as low complement and cryoglobulinemia^13,14^. A higher focus score (the average number of lymphocytic focal infiltrates found per 4 mm^2^ area) in the minor salivary gland biopsy associates with the risk of extra glandular manifestation in SjD, including neurological involvement^15^. Data from the Singapore Sjogren’s syndrome cohort showed that neurological involvement was the most affected internal organ^16^. Both peripheral and central nervous system manifestations are integrated into the EULAR Sjögren’s Syndrome Disease Activity Index (ESSDAI) a tool that evaluates systemic disease complications across 12 domains and serves as a standard metric for assessing treatment response in clinical trials^17^.

## Types/patterns of peripheral nerve involvement in SjD

**Fig. 1:**
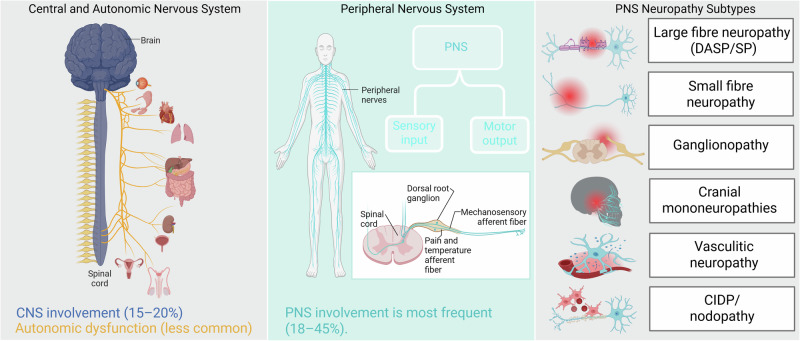
Spectrum of neurological involvement in Sjögren’s disease. Schematic illustrating central, autonomic, and peripheral nervous system manifestations in Sjögren’s disease. Peripheral nerve involvement is the most frequent and includes large-fibre neuropathy, small-fibre neuropathy, sensory ganglionopathy, vasculitic neuropathy, cranial mononeuropathies, and CIDP/nodopathy. The figure highlights the anatomical levels affected and the relative prevalence of CNS, autonomic, and PNS involvement. Created in BioRender. NAYAR, S. (2026) https://BioRender.com/zs2mqki.

There is a wide spectrum of clinical phenotypes represented by the PNS manifestations in SjD (Fig. 1 and Table 2)^18^, which essentially include large and/or small fibre neuropathies, presenting with motor, sensory and/or autonomic symptoms. Most commonly, patients present with tingling, pins and needles, and numbness^19^.

**Table 2:** Pathogenesis, management and treatment of PNS manifestations in SjD

Neuropathy type	Pathogenesis (proposed mechanisms)	Diagnostic test	Treatment SjD related PNS
Large fibre neuropathy DASP SP	Axonal degeneration with features of remyelination, without features of necrotic vascular inflammation^26,30^.	EMG: symmetric axonal pattern of sensory fibre involvement in DASP. reduced sensory nerve action potential and features of acute or chronic denervation in SP. Nerve biopsy: not recommended unless vasculitis is suspected^29^.	1^st^ line: symptomatic treatment such as SNRI, TCA, pregabalin, gabapentin, opioids, botulinum toxin, and capsaicin^74^. 2^nd^ line: IVIg, high dose GC, CyC/RTX, PEX, AZA, MMF high dose IVIg for painful LFN (NCT03700138) or in presence of vasculitic component^19^.
Small fibre neuropathy	Axonal degeneration with reduction of IENFD^36^.	- NCS: normal - Sensory testing (e.g. thermal) or sympathetic sensory testing: abnormal - Evoked potentials: abnormal - Cutaneous biopsy: gold standard for diagnosis showing reduction of IENFD^36^.	1^st^ line: symptomatic treatment. 2^nd^ line: IVIg^81–83^.
Ganglionopathy	- Mononuclear infiltrate^43^ with a prevalent CD8 T cell component^44^. - Autoantibodies attacking the ganglia through fenestrated endothelial cells that form a permeable blood-nerve barrier^37,45,46^.	NCS: reduced or absent sensory nerve action potentials. Somatosensory evoked potential: abnormal.	1^st^ line: IVIg. 2^nd^ line GC. 3^rd^ line: CyC or MMF, RTX, PEX for refractory cases^41,70,73,79,80^.
CIDP	- Impairment regulatory T and B cells^55,58^. - T-lymphocytes directed against the Schwann cell/myelin^55,58^. - Autoantibodies against components of the node of Ranvier^59^.	NCS: abnormal Cutaneous biopsy: evidence of an immune-mediated process.	1^st^ line: IVIg, RTX, AZA, MMF, CyC, GC. 2^nd^ line: RTX. 3^rd^ line: PEX and HSCT in non-responders^54,56,73,87,88^.
Vasculitic Neuropathy	Vasculitis of vasa nervorum involving the peripheral nerves, asymmetrical with loss of myelinated fibres^65^.	Motor and sensory evoked potential: abnormal and involving two or more nerves Cutaneous biopsy: vasculitis^51,65^.	1^st^ line: high dose GC+/− RTX (particularly in presence of cryoglobulinemia); 2^nd^ line: high dose GC+/− CyC or MMF; Maintenance: RTX or MMF or MTX or AZA;^90,93–95,99^.

*AZA* Azathioprine, *CIDP* chronic inflammatory demyelinating polyradiculoneuropathy, *CyC* cyclophosphamide, *DASP* distal axonal sensory polyneuropathy, *EMG* electromyography, *GC* glucocorticoids, *HSCT* hematopoietic stem-cell transplantation, *IENFD* intraepidermal nerve fibre density, *IVIg* intravenous immunoglobulins, *MTX* methotrexate, *MMF* mycophenolate mofetil, *NCS* nerve conductive studies, PEX plasma exchange, *RTX* rituximab, *SP* sensorimotor polyneuropathy, *SNRI* Serotonin-Norepinephrine Reuptake Inhibitors, *TCA* tricyclic antidepressant.

To bring clarity to this complex landscape, a consensus nomenclature for PN manifestations of SjD has been recently published by the Sjogren’s foundation which included a panel of rheumatologist and neurologist experts^20^.

These can be classified clinically, and further defined using neurophysiological studies (NCS), somatosensory evoked potentials (SSEPs) if NCS are normal and pre-ganglionic pathology is suspected, and in some cases peripheral nerve or skin biopsy, the latter to assess for reduced intraepidermal nerve fibre density in SFN.

**Fig. 2:**
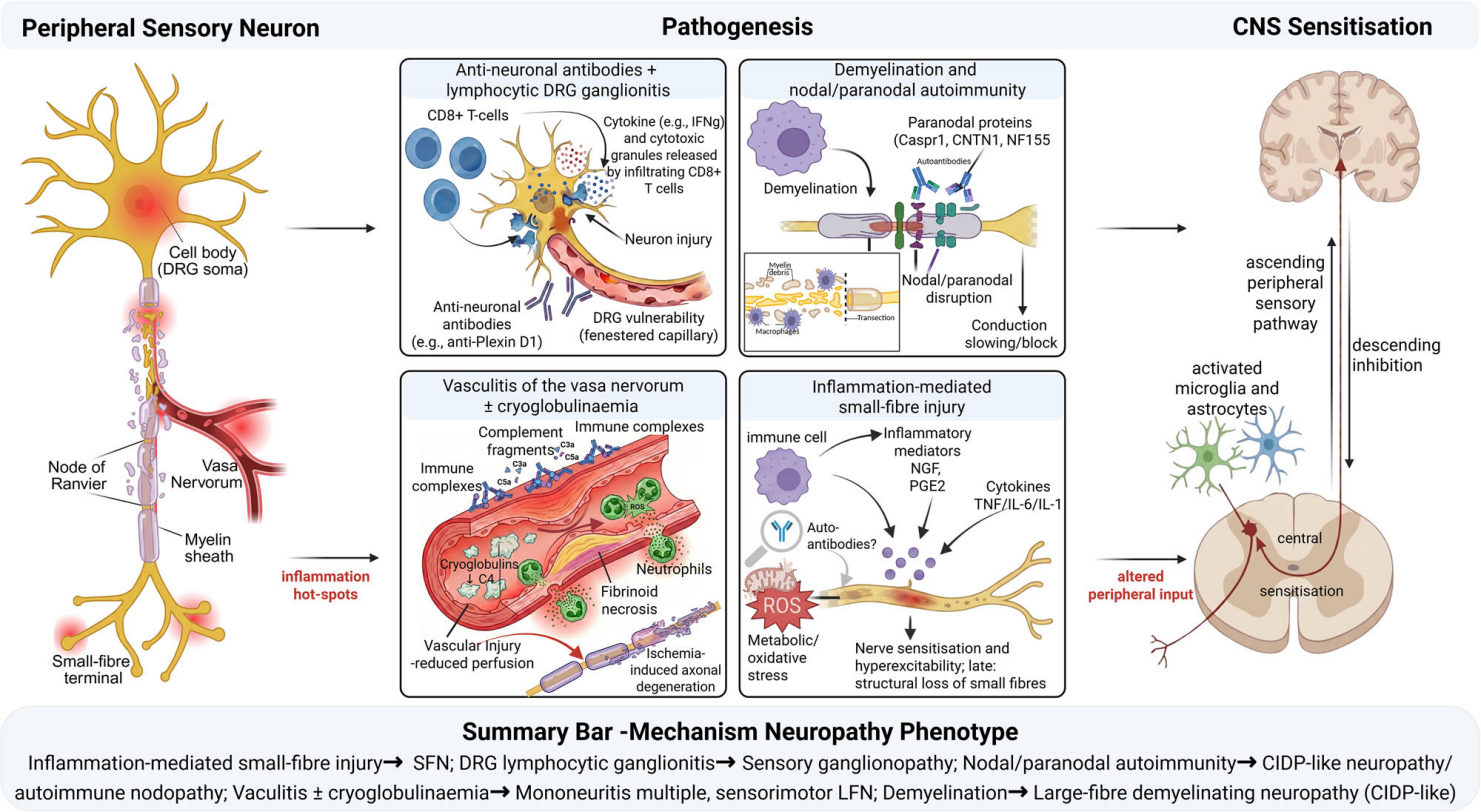
Pathogenic mechanisms underlying peripheral neuropathy in Sjögren’s disease. Integrated mechanistic schematic showing how immune-mediated injury affects distinct regions of the peripheral sensory neuron. CD8⁺ T-cell infiltration and anti-neuronal antibodies cause dorsal root ganglion (DRG) damage and sensory neuron loss. Autoantibodies directed against nodal and paranodal proteins (NF155/186, CNTN1, Caspr1) disrupt axo–glial junctions, resulting in demyelination and conduction block. Vasculitis of the vasa nervorum, driven by immune-complex deposition, complement activation, cryoglobulinemia, and neutrophil-mediated vascular injury, leads to luminal narrowing, reduced perfusion, and ischaemia-induced axonal degeneration. Inflammation and metabolic stress at distal terminals promote small-fibre sensitisation and loss. Abnormal peripheral input enhances activation of microglia and astrocytes within the spinal dorsal horn, contributing to central sensitisation and amplification of pain signalling. Together, these mechanisms give rise to the major neuropathy phenotypes observed in SjD. Created in BioRender. NAYAR, S. (2026) https://BioRender.com/ib4u9m0.

Various pathogenic pathways have been proposed based on clinical findings; these include vasculitis affecting the vasa nervorum and lymphocytic infiltration of the dorsal ganglia, as well as anti-neuronal antibodies^11^ (Fig. 2).

**Fig. 3:**
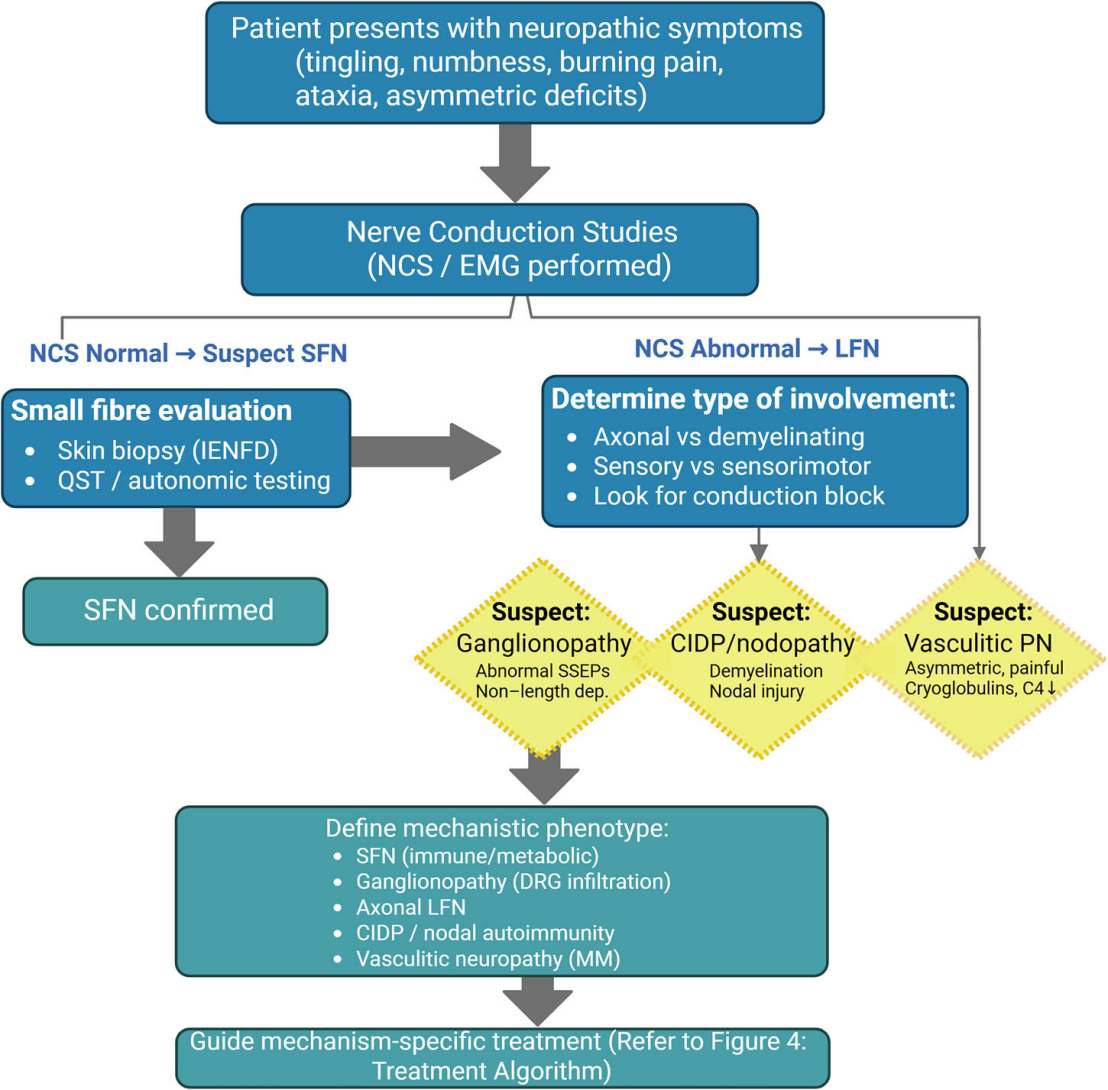
Diagnostic algorithm for peripheral neuropathy in Sjögren’s disease. Flowchart outlining a structured approach for evaluating patients with suspected Sjögren’s-related neuropathy. Initial assessment includes clinical history, neurological examination, and nerve conduction studies (NCS/EMG). Normal NCS directs evaluation toward small-fibre neuropathy using skin biopsy, quantitative sensory testing, or autonomic testing. Abnormal NCS prompts differentiation of axonal, demyelinating, or conduction-block patterns to identify ganglionopathy, nodopathy, vasculitic neuropathy, or large-fibre axonopathy. Mechanistic classification guides treatment selection. Created in BioRender. NAYAR, S. (2026) https://BioRender.com/7f29hy5.

In a multicentric Italian study involving 1695 patients^21^ PNS involvement was accompanied by a more active disease profile and those affected were more frequently treated with immunosuppressants. Sensorimotor polyneuropathies are generally associated with more severe clinical disease, and systemic extra glandular manifestations such as purpura, reduced white blood cells, low complement and cryoglobulinemia; while subjects with pure sensory neuropathy display a milder phenotype^21,22^ (Fig. 3). A case report suggests that immune checkpoint inhibitors used in the cancer field might also trigger neurological manifestations in patients with SjD^23^ as well as metabolic dysregulation^24,25^ and this needs further investigation.

### Large fibre neuropathy (LFN)

Within the large fibre category, there is an even split between purely sensory and sensorimotor presentations. The most frequent manifestation of large fibre neuropathy (LFN) related to SjD is distal axonal sensory polyneuropathy (DASP). The condition involves myelinated Aα and Aβ fibres and often includes some small fibre involvement. While typically bilateral, the distribution can occasionally be asymmetrical^18^. DASP follows a chronic, indolent course, primarily affecting the lower extremities. Patients typically report symmetric paraesthesia, reduced proprioception, and tingling in a characteristic ‘stocking’ distribution. Diagnostic electrophysiology usually confirms a symmetric axonal pattern^26,27^. Nerve biopsy demonstrates axonal loss with no evidence of necrotic vasculitis. It may be accompanied by small fibre neuropathy (SFN). In the latter case symptoms include pain especially at night and at rest, allodynia, itching^28^ and the biopsy may display features of necrotic vascular inflammation of the nerve^29^. Sensorimotor Polyneuropathy (SP) arises when motor fibres are involved alongside sensory ones^30^. Clinical signs include those of DASP plus mild distal muscle weakness (often in the toe or foot extensors) and diminished deep tendon reflexes. In SP, electrodiagnostic studies show reduced action potentials and signs of denervation. Nerve biopsy is recommended only when vasculitis is suspected^31^ and it shows axonal degeneration with remyelination, without necrotic vascular inflammation^32^. Of interest, sensorimotor polyneuropathy could be associated with cryoglobulinemia, low C4 and the development of lymphoma^28,33^. These factors may point toward a possible vasculitic mechanism, which should be considered and investigated where clinically appropriate.

### Small fibre neuropathy (SFN)

SFN has been reported by some studies to affect as many as 30% of patients with SjD^28,34^. The high frequency of SFN in these studies either reflects an ascertainment bias through active screening of patients with SFN for SjD, use of lower specificity tests, differences in referral pathways or cohorts, or the underdiagnosis of SFN elsewhere due to lack of awareness or access to diagnostic tools. SFN can be distal and symmetrical or diffuse. In SFN unmyelinated C and thinly myelinated Aδ fibres are generally affected^18^. Clinically, SFN is characterised by sensory symptoms such as numbness and paraesthesia and prominent neuropathic pain with allodynia (typically burning sensation), but preserved strength and tendon reflexes, joint position and vibration sense; features that would otherwise support LFN. Patients also have reduction in electrochemical skin conductance due to a reduction in sweat gland function^34^ and in some cases restless legs syndrome^27^.

SFN has normally a non–length-dependent distribution, which may allow differentiation from other forms of length-dependent neuropathy, such as from diabetes, which is characterised by a ‘distal-to-proximal’ gradient^35,36^.

Physical examination may reveal allodynia, hyperalgesia, and a reduction in temperature and pinprick sensation. Electrodiagnostic studies are usually normal in patients with SFN. Skin biopsy to assess for reduction in IENFD is the most specific test^36^. Ideally, a diagnosis of SFN should have been ascertained by skin biopsy, however this is rarely done in clinical practice due to poor availability of biopsies and laboratory capability and lack of standardisation in the interpretation. However, Seeliger et al. reported that SjD patients with confirmed SFN had a lower mean IENFD than those with idiopathic SFN, which may reflect differences in the pathogenesis of SFN. Where available, impaired or absent laser-evoked potentials, atypical quantitative sensory testing for thermal stimuli, and/or abnormal sympathetic sensory testing can suggest a SFN diagnosis in accordance with the ESSDAI user guide^17^. Nevertheless, thermal quantitative sensory testing and sympathetic sensory assessments have demonstrated limited specificity and reduced reproducibility^36^. Interestingly in a study comparing 23 patients with SjD and SFN versus 98 patients without SFN, the authors found that the frequency of male patients was higher in the SjD with SFN group, whereas immunological abnormalities (anti-Ro, anti-La, RF, high IgG) were less common. The pathogenetic mechanism has been ascribed to axonal degeneration^22,36^.

### Ganglionopathy

According to a systematic review of SjD-related PN pathology, ganglionopathy accounts for 20% of LFN cases^18^ which seems higher than usually observed in clinical practice. Ganglionopathies represent a form of pure sensory neuropathy resulting from the destruction of neurons within the dorsal root ganglia^37,38^. Sensory neuropathy is usually symmetric. It can occur in a multifocal distribution and is typically non-length dependent. In contrast to distal sensory neuropathy, the symptoms can affect proximal and distal limbs, as well the trunk^39^. Patients classically present with impaired balance due to marked loss of joint proprioception^40,41^ accompanied by numbness and paraesthesia. Clinically there is a reduction in light touch, vibration perception, proprioception and tendon reflexes. The reduction in proprioception leads to involuntary, slow, writhing movements (pseudoathetoid posturing) and inability to perform fine motor tasks. Autonomic dysfunction may occur and, in some cases, ganglionopathy is associated with cerebellar involvement^39^. Electrodiagnostic studies reveal diminished or absent sensory nerve action potentials (SNAPs) in the absence of any motor changes and with preserved needle electromyography^42^. Additionally, abnormalities in somatosensory evoked potentials are frequently noted. While the precise cause remains uncertain, case reports describe a mononuclear cell infiltrate^43^ dominated by CD8^+^ T cells^44^. Other reports indicate that autoantibodies against the ganglia may also be involved^37,38,45,46^. Cases series suggest that many patients with ganglionopathy may be anti-Ro antibody negative with the diagnosis of SjD having been made by salivary gland biopsy^28,32^.

Ganglionopathy not related to SjD can be associated with cancer, especially small cell lung cancer, infections (e.g. HBV), coeliac disease, cancer related medications such as platins and vitamin B6 toxicity^37^.

### Cranial mononeuropathies

Among cranial neuropathies associated with SjD, trigeminal neuropathy is the most frequently reported mononeuropathy, followed by less common involvement of the facial and oculomotor nerves^47,48^; Additional cranial nerves have also been described in isolated cases^14,18,26,28,49,50^. Trigeminal neuropathy is thought to reflect sensory ganglion involvement, in line with the ganglionopathic mechanisms proposed for SjD-related sensory neuronopathy^51^.

Clinically, trigeminal neuropathy usually manifests as a predominantly sensory neuritis, which may be unilateral or bilateral, although unilateral presentations appear more common. In some patients, it occurs as part of a multiple cranial neuropathy syndrome rather than in isolation^52^.

Rarely, optic nerve involvement, including optic atrophy, may represent an early neurologic manifestation of SjD^53^.

### Chronic inflammatory demyelinating polyradiculoneuropathy

CIDP is an uncommon manifestation of peripheral neuropathy in SjD, accounting for less than 1% of reported cases, and in some patients it may precede the diagnosis of SjD^54^.

It typically presents with a slowly progressive course characterized by symmetrical proximal and distal weakness, sensory loss, and reduced reflexes^55,56^.

Increasing recognition of clinical and immunologic heterogeneity suggests that CIDP may include different types of immune-mediated demyelinating neuropathies, and it is unclear if this heterogeneity is also seen in SjD associated CIDP^55^.

Pathologic studies of CIDP during active disease demonstrate inflammatory infiltrates within nerve roots and trunks, with macrophage-mediated demyelination as a hallmark feature. Both cellular and humoral immune mechanisms are implicated, although their relative contributions likely differ across phenotypes. Experimental and clinical evidence supports roles for T-cell responses against myelin antigen^57^, dysregulation of regulatory T- and B-cell compartments, and in a subset of patients, pathogenic antibodies directed against nodal or paranodal protein^55,58^.

Individuals with antibodies against proteins such as CNTN1/CASPR1 or neurofascins are now recognized as having autoimmune nodopathies, which often show a more aggressive onset, atypical features, and reduced responsiveness to conventional CIDP therapies such as IVIg, corticosteroids, or plasma exchange^59^. The recent therapeutic success of FcRn inhibition with efgartigimod further supports an antibody-mediated mechanism in selected cases^60^; although variable response after transition from IVIg indicates ongoing biological heterogeneity^61^.

### Vasculitic neuropathy (VN)

Vasculitic neuropathy in SjD results from immune-mediated inflammation of the vasa nervorum, leading to ischaemic nerve injury. This process may occur in the setting of systemic vasculitic disorders, cryoglobulinemia, or as part of connective tissue disease–associated vasculitis. Clinical clues include painful, asymmetric, rapidly progressive neuropathy, often with normal CSF findings, while nerve biopsy may reveal inflammatory infiltration of vessel walls together with acute or chronic vascular damage. In SjD, this pattern frequently presents as mononeuritis multiplex (MM), a painful asymmetric sensorimotor neuropathy caused by involvement of multiple individual nerve trunks^51,62^.

Although initially multifocal and patchy, disease progression may lead to more diffuse and apparently symmetric deficits^63^ especially when associated with vasculitis^64^. Cases of aseptic meningitis followed by mononeuritis multiplex have also been reported in SjD^62^.

Electrophysiologic evaluation typically demonstrates marked side-to-side asymmetry of motor and sensory responses, while EMG often supports an axonal pattern of injury^65^. Conduction block may be present in selected cases and can aid diagnostic classification. Nerve biopsy remains highly informative when vasculitis is suspected^66,67^.

The mechanisms underlying MM in SjD remain incompletely defined. Some reports have identified anti-GM1 antibodies, raising the possibility of complement-mediated or antibody-mediated motor axonal dysfunction and focal conduction failure in a subset of patients^67^. Several studies have characterised MM in SjD^18,68–70^ or in overlap with other autoimmune disorders^69^. A study from Mori et al., identified 11 out of 96 patients with SjD affected by MM. In all the patients ANCA antibodies and cryoglobulins were not detected^51^.

## Treatment of PNS manifestations in SjD

PNS manifestations are clinical complications detected in SjD and often precede the onset of sicca symptoms. In some cases, such as sensory ganglionopathy, they may evolve over days/weeks and there may be a small window of opportunity to improve outcomes^71^. Beyond this point, the inflammatory reaction likely subsides, leaving irreversible damage, and treatment becomes ineffective. That being said, the only study of sensory ganglionopathy with prospective follow-up suggested a slowly progressive course over years^72^ suggesting a difference in presentation with that seen in some other settings and the potential for an effective treatment to avert further damage. Early diagnosis and prompt initiation of treatment, where appropriate, is paramount in order to obtain clinical response.

**Fig. 4:**
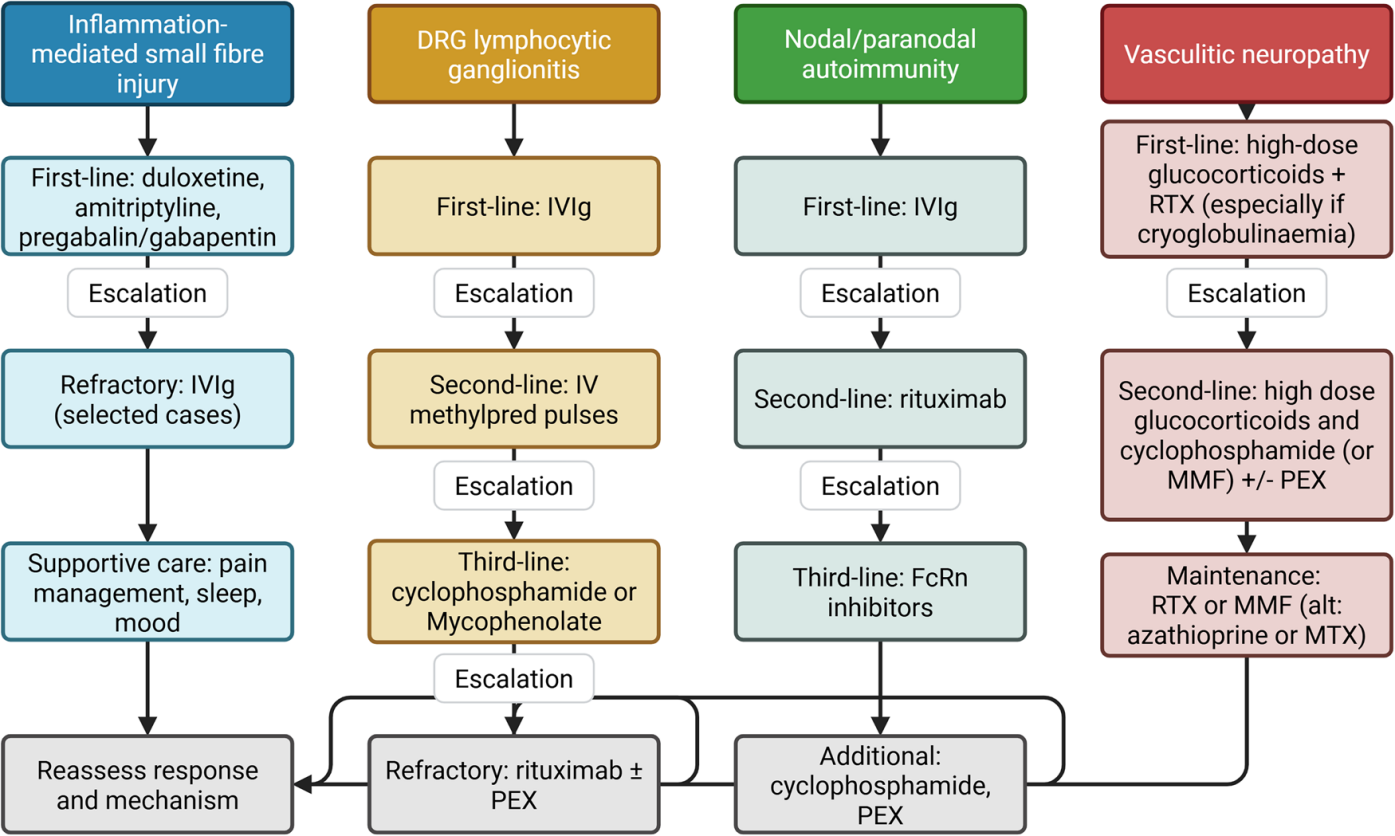
Mechanism-based treatment strategies for peripheral neuropathy in Sjögren’s disease. Treatment pathways organised by pathogenic mechanism. Small-fibre neuropathy is managed with symptomatic analgesic agents and IVIg in selected cases. DRG ganglionitis is treated with IVIg followed by corticosteroid pulses, cyclophosphamide, or rituximab/plasma exchange for refractory disease. Nodal/paranodal autoimmunity is treated with IVIg, rituximab, and emerging FcRn inhibitors. Vasculitic neuropathy requires high-dose glucocorticoids with rituximab or cyclophosphamide, particularly in the presence of cryoglobulinaemia, followed by maintenance immunosuppression. The algorithm emphasises reassessment of therapeutic response and mechanism. Created in BioRender. NAYAR, S. (2026) https://BioRender.com/6r4gnlp.

According to the 2020 EULAR guidelines treatment of PNS involvement in SjD includes high doses of corticosteroids (GC), intravenous immunoglobulins (IVIg), rituximab (RTX), plasma exchange (PEX) and cyclophosphamide (CyC)^73^. However, the specific treatment needs to be tailored based on the underlying pathogenetic mechanism (Fig. 4).

### Distal axonal sensory polyneuropathy (DASP)

When Distal Axonal Sensory Polyneuropathy (DASP) presents with purely sensory features, management focuses on alleviating neuropathic pain^74^. A 2019 review within the neurology field identified drugs targeting neuropathic symptoms such as serotonin-norepinephrine reuptake inhibitors (SNRIs), gabapentinoids (pregabalin/gabapentin), and tricyclic antidepressants (TCAs), as good therapeutic options and first-line therapies, while topical treatments such as lidocaine, capsaicin, and tramadol were assigned as second-line options. Potent opioids and botulinum toxin A were relegated to third-line status^74^.

In more severe cases, particularly those involving motor nerve impairment or systemic SjD activity, immunomodulatory treatments such as intravenous immunoglobulin (IVIg), high-dose GC (e.g., methylprednisolone pulses), CyC, azathioprine (AZA), mycophenolate mofetil (MMF), PEX, RTX have shown clinical benefit^22^. GC, IVIg, PEX, RTX, and sirolimus have been used in patients with sensory neuronopathy secondary to cancer^75,76^. The detection of sensorimotor polyneuropathy should prompt evaluation for cryoglobulinaemic vasculitis, the management of which is reported in Section “Vasculitis neuropathy (VN)”^2^. A French multicentre retrospective analysis of 40 SjD patients with biopsy-confirmed neuropathy revealed that those without evidence of vasculitis (*n* = 18) rarely responded to immunosuppressants (0% vs 55%), with positive responses correlating more with necrotizing than lymphocytic vasculitis^77^. Conversely, a retrospective series of 19 SjD patients lacking necrotizing vasculitis suggested a potential clinical benefit from IVIg^78^. A small single-centre clinical trial testing the benefit of IVIg in patients with SjD and painful large fibre sensory polyneuropathy without diagnosis of SFN is currently ongoing and could potentially inform treatment choices (TINISS; NCT03700138).

### Ganglionopathy

Ganglionopathy is a distinctive PNS manifestation in SjD typically presenting with severe sensory ataxia (poor muscle control that causes clumsy movements) and significant functional impairment. In SjD-related ganglionopathy IVIg is the first line treatment according to the EULAR guidelines followed by methylprednisolone pulses as second line. CyC is generally reserved for refractory cases^73^. PEX and RTX have also been successfully used in patients unresponsive to standard regimens^70,79,80^. In a study of 15 patients by Font et al., treatments included GC (*n* = 13), CyC (*n* = 4) and IVIg (*n* = 1) but most patients slowly progressed despite treatment. Interestingly, in a retrospective study including 13 SjD patients affected by ganglionopathy, patients were treated with different immunosuppressants including GC, MMF, hydroxychloroquine, IVIg and CyC^41^. Improvement or stability was seen in half of the patients treated with IVIg (3 out of 6) but in the majority of those treated with MMF alone or in combination with other medications (6 out of 7)^41^.

### SFN

Typical first line approaches to SFN are neuroactive medications such as low dose tricyclic antidepressants (TCAs), duloxetine or pregabalin. Opioid analgesics are also a treatment option in patients with SjD related SFN^36^. However, these medications can be associated with exacerbation of dryness, particularly seen with TCAs, and fatigue. Morozumi described a case series of 5 patients with clinical features of SFN supported by reduction in small fibres in sural nerve biopsy whose symptoms failed to respond to neuroactive medications but who all had a seemly rapid improvement in pain VAS following IVIg^81^. A similar case reported by Wakasugi et al. with diagnosis confirmed by reduction in epidermal nerve fibre density on skin biopsy also responded rapidly to IVIg^82^. A retrospective French case series of 12 patients with SjD (8 of whom also had other concomitant CTD diagnoses) and skin biopsy-confirmed SFN refractory to conventional treatments were also successfully treated with IVIg. Remission was achieved and maintained after stopping the treatment in 9 patients with improvement of several neurological and health quality scores^83^. However, given the retrospective open-label nature of these studies and the high cost and limited availability of IVIg, higher quality evidence is needed. A randomised controlled trial of IVIg in idiopathic SFN neuropathy was negative, although this might reflect a differing underlying pathology^84^. Physical exercise may be beneficial in SFN associated to diabetes^85^; in SFN secondary to sarcoidosis, a drug called ARA-290 (Cibinetide) has shown positive results in a clinical trial^86^.

### Chronic inflammatory demyelinating polyradiculoneuropathy (CIDP)

For CIDP in the context of SjD, EULAR recommendations advise IVIg as first line, RTX as second line, and PEX or CyC as rescue treatment^73,87,88^. Similar options are used in CIDP not related to SjD and short courses of GC, IV or oral, are often used at initiation. MMF is also used in clinical practice despite limited evidence^89^. Recently, the FcRn inhibitor efgartigimod alfa has been approved by the FDA for CIPD and as monotherapy by the EMA for progressive or relapsing active CIDP following treatment with GC or IVIg^60^.

### Vasculitis neuropathy (VN)

Current therapeutic protocols for Vasculitic Neuropathy (VN) lack validation from randomized controlled trials, relying instead on retrospective data and expert consensus^90–92^ with some studies including patients both with and without systemic vasculitis. Moreover, outcome measures differed across studies^93–95^. Glucocorticoids (GC) remain the primary first-line intervention, though clinical relapse often necessitates the addition of steroid-sparing agents like Azathioprine (AZA) or Cyclophosphamide (CyC)^94,95^.

In a large retrospective cohort study including 60 patients with VN, AZA was successfully used to obtain remission in 41% of patients. However most of the patients still required the addition of another immunosuppressant such as CyC due to disease recurrence^95^.

The 2010 Peripheral Nerve Society guidelines provided Good Practice Point recommendations for treating non-systemic VN^94^.

Treatment is indicated for all patients with progressive VN or biopsy-proven active vasculitis. While GC monotherapy is generally preferred, combination therapy (GC with CyC, MTX, or AZA) is reserved for patients with rapidly progressing VN or those non-responsive to corticosteroids alone. For severe cases of VN, the guidelines favour CyC. Upon reaching remission, prednisone may be discontinued or maintained at a low dosage alongside AZA or MTX^94^. High-dose GC (prednisone at 1 mg/kg/day) combined with CyC (administered orally or intravenously) is the preferred treatment for SjD patients exhibiting multiple mononeuropathies^96^. Notably, SjD patients with mononeuritis multiplex have demonstrated favourable responses to CyC^97^. Furthermore, RTX has proven effective for SjD patients with vasculitis-related PNS involvement^80^. According to the 2020 EULAR SjD management guidelines, GC are recommended as first-line therapy, followed by oral immunosuppressants or RTX as second-line options, and CyC pulses with or without plasma exchange as third-line intervention^98^. RTX and GC are the first line treatment in presence of cryoglobulinemia^99^. Overall, treatment strategies for peripheral neuropathies in SjD depend on the underlying mechanism whether primarily immune-mediated, vasculitic, or degenerative. While IVIg, GC, and immunosuppressants such as MMF and RTX are commonly used, the evidence base varies considerably between neuropathy subtypes. Future research could also explore the role of immunometabolic dysregulation in SjD-associated neuropathies. Targeting these pathways could open new therapeutic avenues, particularly in neuropathy subtypes resistant to conventional immunosuppressive therapy. Prospective, controlled studies remain urgently needed to inform optimal therapeutic approaches.

## Discussion and clinical trial design considerations

**Fig. 5:**
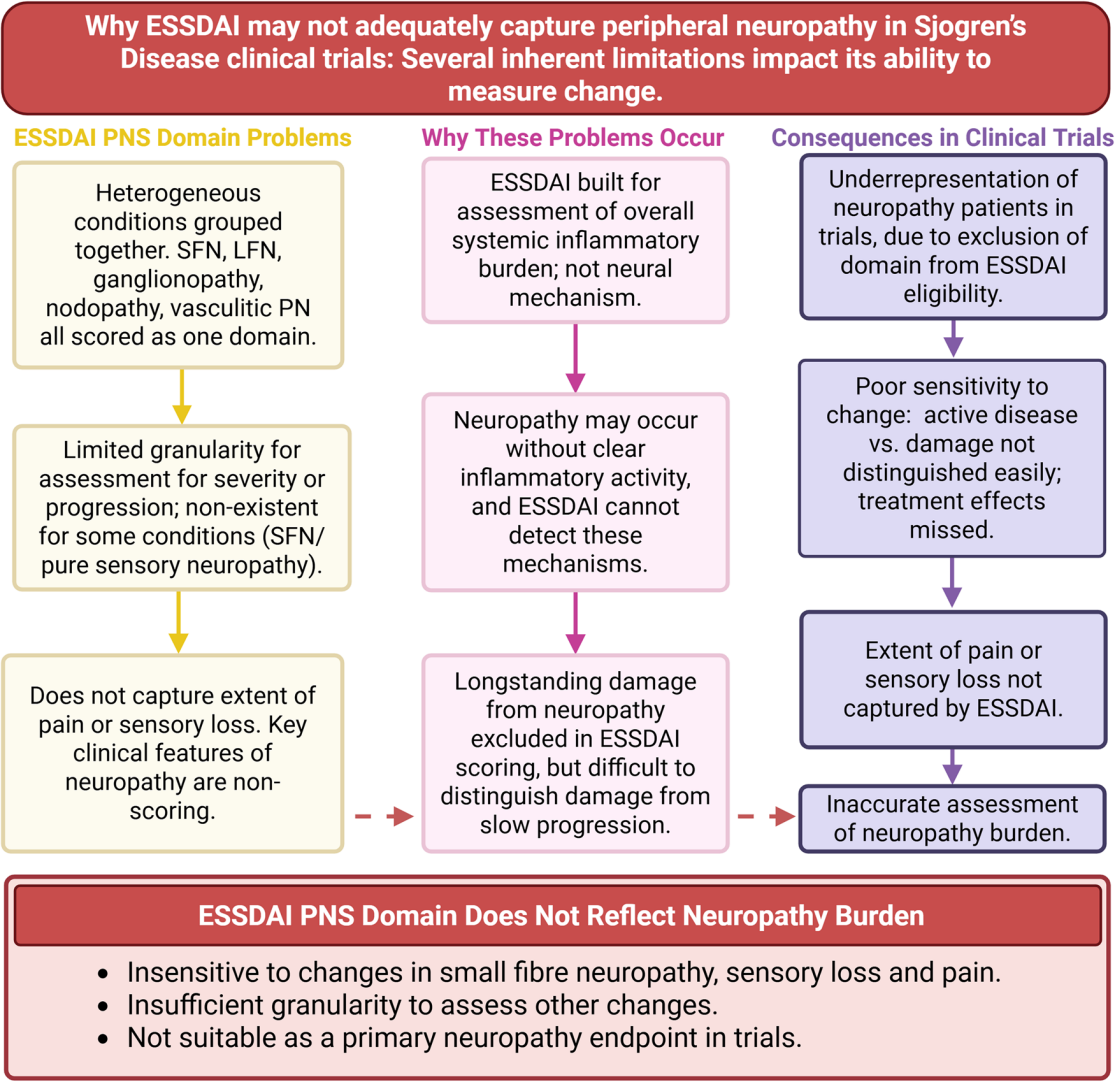
Limitations of ESSDAI for capturing peripheral neuropathy in Sjögren’s disease. Flowchart summarising structural limitations of the ESSDAI PNS domain. Heterogeneous neuropathies are grouped under a single category, severity grading is limited, and key symptoms such as pain, sensory loss, and ataxia are not scored. There are challenges in clinically distinguishing active immune-mediated injury from longstanding damage, reducing sensitivity to clinical change. These issues contribute to underrepresentation of neuropathy in clinical trials and may limit detection of meaningful treatment effects. Created in BioRender. NAYAR, S. (2026) https://BioRender.com/ikf3q45.

Most contemporary clinical trials use the ESSDAI as the primary outcome measure. The ESSDAI is based on 12 organ-specific domains, each with 3–4 levels that are weighted according to the importance of the domain involvement. The development and validation of the ESSDAI was a significant advance that encouraged clinical trial activity in SjD. Several phase 2 randomised controlled trials have shown superiority of novel intervention over placebo using ESSDAI as their primary outcome^100–107^ and several of these drugs have now progressed into phase 3 clinical trials. However, use of the PNS domain of the ESSDAI poses several challenges (Fig. 5). Firstly, there are heterogeneous PNS manifestations distributed across different activity levels meaning that the scale is more nominal than ordinal. For example, pure sensory neuropathy only occurs in low disease activity whereas CIDP occurs in moderate or high activity levels depending on degree of ataxia or motor impairment. Secondly, even if a relevant immune mediated process is switched off by an intervention, it is possible that symptoms may persist due to irreversible nerve damage. The ESSDAI allows for scoring stable non-progressive disease for 12 months as no activity, but phase 2 trials commonly do not last this long. A physician might consider scoring ‘no activity’ if there has been a rapidly evolving neuropathy that then stabilises after intervention, but this clinical presentation may be challenging to recruit to a placebo-controlled trial. Further, the PNS domain is one of the most highly weighted domains, meaning that challenges in objectively assessing improvement could impact overall trial outcomes (Fig. 5). Consequently, several clinical trials have not allowed PNS domain activity (alongside that of other highly weighted domains) to count towards the ESSDAI eligibility threshold for inclusion, to avoid recruiting patients whose only activity is in the PNS domain and to increase the frequency of lower weighted domains that are perceived to be more sensitive to change. One result of this might be the underrepresentation of PNS involvement in clinical trials and this, together the challenges of scoring, may make it difficult to understand if successful phase 3 drugs are also capable of improving peripheral neuropathy in SjD. The fact that the different PNS manifestations might have subtle difference in immunopathogenesis, as alluded to in this review, only complicates this further.

Importantly, the limitations of using ESSDAI scoring as an inclusion criterion in randomized controlled trials also contributes to the underrepresentation of patients with high symptom burden but low systemic inflammatory activity. This includes the so-called “population 2” phenotype, characterized by prominent sicca symptoms, chronic fatigue, and pain-often of neuropathic origin-despite minimal ESSDAI elevation. These individuals are frequently excluded from clinical trials, despite experiencing substantial functional impairment and significantly reduced quality of life. In many cases, neuropathic pain is a major contributor to their disease burden. The exclusion of this phenotype not only limits the generalizability of trial outcomes but may also obscure treatment effects that are meaningful to patients but are not adequately captured by current systemic activity indices.

We would therefore advocate consideration of bespoke clinical trials targeting specific PNS manifestations once we have drugs with proven efficacy in systemic SjD. This is particularly relevant for sensory neuropathy and ganglionopathy where we have no clear evidence base for management. Although optimising treatments for vasculitic neuropathies would also provide benefits, as least we currently have some immunosuppressive approaches that are used to good effect in clinical practice.

**Fig. 6:**
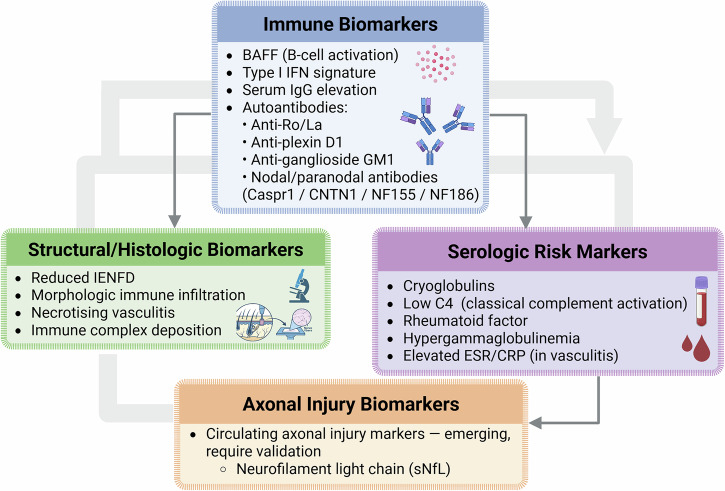
Biomarker landscape supporting mechanistic classification of neuropathy in Sjögren’s disease. Overview of immune, structural, serologic, electrophysiologic, and axonal injury biomarkers used to evaluate neuropathy mechanisms. Immune biomarkers include BAFF, type I interferon signatures, serum IgG elevation, and neuronal/nodal autoantibodies. Structural biomarkers include reduced intraepidermal nerve fibre density, DRG infiltration, necrotising vasculitis, and immune-complex deposition. Serologic risk markers include cryoglobulins, low complement, rheumatoid factor, and hypergammaglobulinemia. Electrophysiologic measures (axonal loss, demyelination, conduction block, abnormal SSEPs) help differentiate neuropathy subtypes. Emerging axonal injury markers such as serum neurofilament light chain (sNfL) may offer sensitive measures of nerve damage and mechanistic activity. Created in BioRender. NAYAR, S. (2026) https://BioRender.com/sammp8b.

Selection of suitable outcome measures is critical to the success of clinical trials (Fig. 6). In this regard, in a 2018 study we found a statistically significant association between B cell markers such as BAFF and the PNS domain (*p* < 0.002) in the sera of 553 SjD patients belonging to the UK primary Sjögren’s syndrome registry (UKPSSR) Biobank^108^. This suggests that domain specific trials are warranted. The only existing trial in SjD neuropathy to our knowledge is a placebo-controlled trial of IVIg in non-vasculitic large-fibre neuropathy (TINISS; NCT03700138). The co-primary outcomes of this trial are 20% improvement in numerical pain scale and the Rasch-built Overall Disability Scale (R-ODS)^109^. Whilst improvements in patient-reported outcomes (PROs) are clearly important, many contemporary trials have reported large placebo responses making detection of drug efficacy challenging, and means to limit these responses by, for example, run-in periods, should be considered. R-ODS, like other peripheral neuropathy measures, has not been validated in SjD. Depending on the context, other measures could be considered such as the Utah early neuropathy scale, skin biopsy, quantitative threshold testing and other symptom scores such as the Quality of life questionnaire to assess chemotherapy induced peripheral neuropathy 20 (QLQ-CIPN20) or its shorter forms^110,111^. The value of high sensitivity assays for biomarkers of damage such as serum neurofilament light chain should also be explored in SjD^111,112^.

## Outlook

Neurological manifestations frequently occur in SjD, often predating the primary diagnosis. However, establishing precise prevalence remains challenging due to clinical heterogeneity and cohort ascertainment biases. While vascular, ischaemic, and immunological pathways have been identified, the exact pathogenic mechanisms for most neurological involvement remain elusive. Given the impact of PNS involvement upon quality of life, there is a critical need to further characterise pathogenic mechanisms and undertake randomized controlled trials to better guide therapeutic strategies.

## Supplementary information

Transparent Peer Review file

## Data Availability

This review article contains no original research data.
