# Supplementary material for: Clinical features and pathogenesis of Sjogrens disease related peripheral neuropathy and their relevance to clinical trials
Source: Commun Med (Lond). 2026 Jun 19;6:350. doi: 10.1038/s43856-026-01688-2 (PMC13282401; doi:10.1038/s43856-026-01688-2)
Supplement: Supplementary file 1 — Transparent Peer Review file [file 43856_2026_1688_MOESM1_ESM.pdf]

nature portfolio

Peer Review File

Clinical features and pathogenesis of Sjogrens Disease Related Peripheral Neuropathy and their relevance to clinical trials

Corresponding Author: Professor Benjamin Fisher

This file contains all reviewer reports in order by version, followed by all author rebuttals in order by version.

Version 0:

Reviewer comments:

Reviewer #1

(Remarks to the Author)
In this review, the authors report that neurologic involvement is a common complication of Sjögren’s disease (SjD), affecting up to 20% of patients. It can impact the central, peripheral, and autonomic nervous systems, with the peripheral nerves being most frequently affected. The wide range of neurologic symptoms makes diagnosis and treatment challenging, and management should target the underlying neuropathologic mechanisms, which are often unclear. The authors focus on peripheral nerve manifestations, explore shared and distinct pathogenic pathways, and discuss their implications for diagnosis and treatment.
Introduction: It is well summarized and well documented.
Line 48: After “lymphoma” there should be a period.
Line 91 – 3. Types/Patterns of Peripheral Nerve Involvement in SjD:
• The figure and table are well presented.
Line 110: “The presence of purpura, extra-glandular manifestations, leukopenia, low complement, and cryoglobulinemia principally characterized patients with sensorimotor polyneuropathies, while subjects with pure sensory neuropathy displayed a milder phenotype.” → Note: purpura is already considered an extra-glandular manifestation.
Line 152: “non coma” appears at the end of the phrase—needs correction.
Line 269 – 4. Treatment of PNS Manifestations in SjD:
• Figure 4 provides a logical and well-designed representation of treatment.
Line 392 – 5. Discussion and Clinical Trial Design Considerations:
• It is well known that the ESSDAI is useful but has limitations, especially for peripheral neuropathy.
• Unfortunately, PNS involvement is underrepresented in clinical trials, which may overlook patient populations with a high symptom burden but low systemic inflammation.
• There is a clear need for tailored clinical trials targeting specific PNS manifestations.
• Outcome measures and biomarkers should be carefully selected, combining clinical, functional, and patient-reported metrics.
• A major challenge is that placebo effects and irreversible nerve damage can complicate trial design.
Line 504 – References: The references provide good coverage of the literature, including studies, case reports, and reviews.
Overall, the impression of this review is positive, and I am optimistic that the authors will continue with clinical studies.

Reviewer #2

(Remarks to the Author)
This article is particularly relevant for clinicians. Neurological manifestations of Sjögren's disease are likely to delay the diagnosis of Sjögren's disease. The article summarizes evidence-based data on peripheral nervous system damage in various forms of Sjögren's disease.
I have no comments on the shortcomings of this article.

Version 1:

Reviewer comments:

Reviewer #1

(Remarks to the Author)
I have no comments on the shortcomings of the new version of this article.

Open Access This Peer Review File is licensed under a Creative Commons Attribution 4.0 International License, which permits use, sharing, adaptation, distribution and reproduction in any medium or format, as long as you give appropriate credit to the original author(s) and the source, provide a link to the Creative Commons license, and indicate if changes were made.
In cases where reviewers are anonymous, credit should be given to 'Anonymous Referee' and the source.
The images or other third party material in this Peer Review File are included in the article's Creative Commons license, unless indicated otherwise in a credit line to the material. If material is not included in the article's Creative Commons license and your intended use is not permitted by statutory regulation or exceeds the permitted use, you will need to obtain permission directly from the copyright holder.
To view a copy of this license, visit https://creativecommons.org/licenses/by/4.0/

Point by point reply

Dear Editor,

We sincerely thank you and the reviewers for the time and effort spent evaluating our manuscript. We appreciate the constructive feedback and have carefully considered each comment. Below, we provide a detailed, point-by-point response to address all concerns raised. Changes in the revised manuscript (Pucino et al_PND in SjD_track-changes) are clearly highlighted.

In addition to the reviewers' comments, we have slightly modified the abstract adding a short introduction on Sjogren's disease as per Editor's suggestion. We have also changed one of the proposed potential outcomes for a PNS trial to the 'Utah Early Neuropathy Scale' on line 457 as, depending on the clinical context, this may be more appropriate to Sjogren's and we have made a couple of typographical changes to the ESSDAI figure (Figure 5 – has replaced the original one in the re-submitted version)

We believe that the revisions have strengthened the manuscript and addressed all reviewer concerns. We appreciate your consideration and look forward to your decision.

Sincerely,
Prof Benjamin Fisher

Reviewer 1

In this review, the authors report that neurologic involvement is a common complication of Sjögren's disease (SjD), affecting up to 20% of patients. It can impact the central, peripheral, and autonomic nervous systems, with the peripheral nerves being most frequently affected. The wide range of neurologic symptoms makes diagnosis and treatment challenging, and management should target the underlying neuropathologic mechanisms, which are often unclear. The authors focus on peripheral nerve manifestations, explore shared and distinct pathogenic pathways, and discuss their implications for diagnosis and treatment.
Introduction: It is well summarized and well documented.
Line 48: After "lymphoma" there should be a period.
Line 91 – 3. Types/Patterns of Peripheral Nerve Involvement in SjD:
• The figure and table are well presented.
Line 110: "The presence of purpura, extra-glandular manifestations, leukopenia, low complement, and cryoglobulinemia principally characterized patients with sensorimotor polyneuropathies, while subjects with pure sensory neuropathy displayed a milder phenotype." → Note: purpura is already considered an extra-glandular manifestation.
Line 152: "non coma" appears at the end of the phrase—needs correction.
Line 269 – 4. Treatment of PNS Manifestations in SjD:
• Figure 4 provides a logical and well-designed representation of treatment.
Line 392 – 5. Discussion and Clinical Trial Design Considerations:
• It is well known that the ESSDAI is useful but has limitations, especially for peripheral neuropathy.
• Unfortunately, PNS involvement is underrepresented in clinical trials, which may overlook patient populations with a high symptom burden but low systemic inflammation.
• There is a clear need for tailored clinical trials targeting specific PNS manifestations.
• Outcome measures and biomarkers should be carefully selected, combining clinical,

functional, and patient-reported metrics.
• A major challenge is that placebo effects and irreversible nerve damage can complicate trial design.
Line 504 – References: The references provide good coverage of the literature, including studies, case reports, and reviews.
Overall, the impression of this review is positive, and I am optimistic that the authors will continue with clinical studies.

We thank the reviewer for his/her appreciation and the positive comments on this manuscript. We have amended the text accordingly.
- Line 48: After "lymphoma" there should be a period. Now corrected in Line 52 of the document Pucino et al_track-changes.
- Line 91 – 3. Types/Patterns of Peripheral Nerve Involvement in SjD:
The figure and table are well presented. Thank you very much for your comment.
- Line 110: "The presence of purpura, extra-glandular manifestations, leukopenia, low complement, and cryoglobulinemia principally characterized patients with sensorimotor polyneuropathies, while subjects with pure sensory neuropathy displayed a milder phenotype." Note: purpura is already considered an extra-glandular manifestation.
We have amended the text as follows: "The presence of extra-glandular manifestations (including lower limb purpura) in association with leukopenia". Line 114 of the document Pucino et al_track-changes
Line 152: "non coma" appears at the end of the phrase—needs correction. Corrected in Line 160.
Line 269 – 4. Treatment of PNS Manifestations in SjD:
• Figure 4 provides a logical and well-designed representation of treatment. Many thanks for your comment.
Line 392 – 5. Discussion and Clinical Trial Design Considerations:
• It is well known that the ESSDAI is useful but has limitations, especially for peripheral neuropathy.
• Unfortunately, PNS involvement is underrepresented in clinical trials, which may overlook patient populations with a high symptom burden but low systemic inflammation.
• There is a clear need for tailored clinical trials targeting specific PNS manifestations.
• Outcome measures and biomarkers should be carefully selected, combining clinical, functional, and patient-reported metrics.
• A major challenge is that placebo effects and irreversible nerve damage can complicate trial design.
The discussion covers all the points highlighted by the reviewer.
Line 504 – References: The references provide good coverage of the literature, including studies, case reports, and reviews. We thank the reviewer for his/her comment.

Reviewer 2:

This article is particularly relevant for clinicians. Neurological manifestations of Sjögren's disease are likely to delay the diagnosis of Sjögren's disease. The article summarizes evidence-based data on peripheral nervous system damage in various forms of Sjögren's disease.
I have no comments on the shortcomings of this article

We thank the reviewer for his/her appreciation and the positive comments on this manuscript.
